# Aging of Liver in Its Different Diseases

**DOI:** 10.3390/ijms232113085

**Published:** 2022-10-28

**Authors:** Tijana Radonjić, Marija Dukić, Igor Jovanović, Marija Zdravković, Olga Mandić, Višeslav Popadić, Maja Popović, Novica Nikolić, Slobodan Klašnja, Anica Divac, Zoran Todorović, Marija Branković

**Affiliations:** 1 University Hospital Medical Center Bežanijska Kosa, 11000 Belgrade, Serbia; 2 Faculty of Medicine, University of Belgrade, 11000 Belgrade, Serbia

**Keywords:** cell, senescence, SASP, DDR, inflammaging, liver, elderly

## Abstract

The proportion of elderly people in the world population is constantly increasing. With age, the risk of numerous chronic diseases and their complications also rises. Research on the subject of cellular senescence date back to the middle of the last century, and today we know that senescent cells have different morphology, metabolism, phenotypes and many other characteristics. Their main feature is the development of senescence-associated secretory phenotype (SASP), whose pro-inflammatory components affect tissues and organs, and increases the possibility of age-related diseases. The liver is the main metabolic organ of our body, and the results of previous research indicate that its regenerative capacity is greater and that it ages more slowly compared to other organs. With age, liver cells change under the influence of various stressors and the risk of developing chronic liver diseases such as non-alcoholic fatty liver disease (NAFLD), non-alcoholic steatohepatitis (NASH), alcoholic steatohepatitis (ASH) and hepatocellular carcinoma (HCC) increases. It has been proven that these diseases progress faster in the elderly population and in some cases lead to end-stage liver disease that requires transplantation. The treatment of elderly people with chronic liver diseases is a challenge and requires an individual approach as well as new research that will reveal other safe and effective therapeutic modalities.

## 1. Introduction

**Figure 1:**
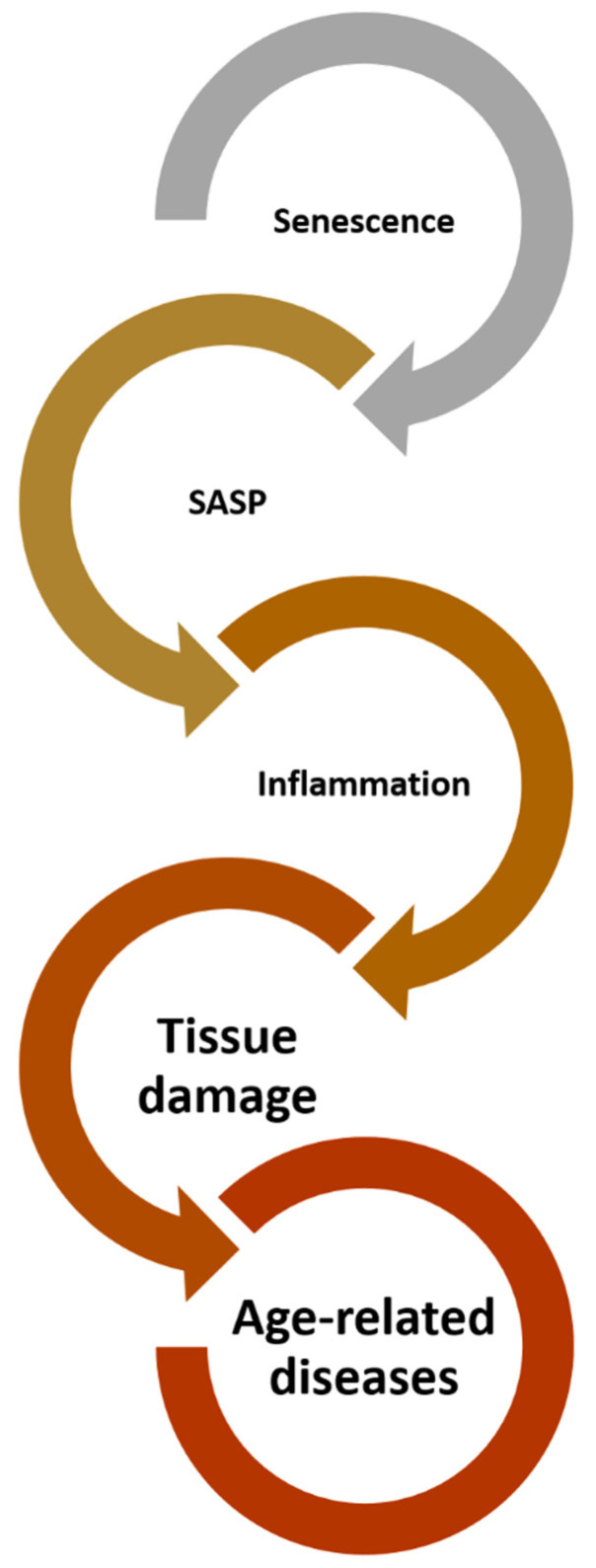
From senescence to age-related diseases. Senescence through components of senescence-associated secretory fenotype (SASP) causes inflammation in tissues and organs which in the end results in occurrence of age-related diseases.

According to the results of recent research, the world population is getting older. The incidence of many diseases increases with age, and the basis of some of them may be changes which are associated with aging [1,2]. That is why we call them age-related diseases. This is one of the reasons why the number of investigations of cellular senescence is rising (Figure 1) [3].

The first studies of cellular senescence date back to the middle of the last century [3,4]. Cellular senescence is a permanent cell cycle arrest that reduces the proliferative and regenerative capacity of cells [5]. It can be caused by various factors such as telomere dysfunction, deoxyribonucleic acid (DNA) damage, oxidative stress, oncogenic activity, etc. [5]. The most common cause of the induction of senescence is the activation of a DNA-damage response [6]. The metabolism of senescent cells is distinct from non-senescent cells and they are metabolically active regardless of the cell cycle arrest [7]. These phenotypically altered cells secrete various molecules, primarily cytokines, chemokines and proteases which make the senescence-associated secretory phenotype (SASP). SASP has numerous roles in the human body and its components affect surrounding cells paracrine [8]. These molecules have been detected in numerous studies, mostly in vitro, and can serve as markers of senescence [3]. The determination of these markers can help in the recognition of senescent cells, as well as in a potential therapeutic approach to age-related diseases [1,9]. Considering that in these conditions the production of pro-inflammatory cytokines is increased, the term “inflammaging” was introduced into the literature [1,8]. The consequences of cell aging are present in all tissues and organs and progress at different speeds depending on the type of tissue or organ, gender and the effects of endogenous and exogenous factors. The liver is a metabolic and endocrine active organ that is often called “the laboratory of the organism” and the first research on the topic of its aging dates back to the end of the last century [10]. It has been proven that the incidence of liver diseases, as well as mortality from these diseases, increases with age [11]. Age is often an independent factor of poor outcomes in liver diseases [12]. In this connection, the pathophysiology of non-alcoholic fatty liver disease (NAFLD), non-alcoholic steatohepatitis (NASH), alcoholic steatohepatitis (ASH), viral hepatitis and hepatocellular carcinoma (HCC) in elderly patients was examined [12,13,14]. Based on the results of these studies, it was concluded that due to proven differences between younger and older people with these diseases, the treatment of patients must be approached individually.

## 2. Cellular Senescence and Its Characteristics

In the 1960s, Hayflick and Moorhead were the first to describe the concept of cellular senescence by observing human diploid fibroblasts [3,4]. Considering the importance of such discovery, researchers from different fields of natural sciences tried to investigate this phenomenon in more detail. It has been shown that cellular senescence is related to diseases that accompany aging, so new achievements in this area could enable the prevention of these diseases, better survival and longer life expectancy of the people [3]. Cellular senescence corresponds to an irreversible stable cell cycle arrest that limits cell proliferation and promotes chronic inflammation [4]. Still, it is not known if the cellular senescence can, potentially, be reversible, but if confirmed, this would be of great importance especially in oncology as senescent cells appear to be particularly important for relapse of malignancies. As a response to various forms of chemotherapy, cells enter senescence. However, it has been shown that tumor cells are able to avoid that fate by different mechanisms. In this way, they manage to enter a specific state of rest and to live longer despite the use of chemotherapeutic agents, which increases the possibility of recurrence of the malignant disease [15,16]. An example from clinical practice is the ability of some breast cancer cells to avoid the effect of an adequate concentration of doxorubicin, as well as the ability of some lung cancer cells to bypass the entry into senescence caused by the administration of camptothecin [17,18]. Studies have shown that the cell’s ability for this process depends on the expression of the cyclin-dependent kinase [15]. On the other hand, Zampetidis et al. are of the opinion that the possibility of avoiding entry into senescence is a consequence of genome instability [19]. Senescence represents the cell’s response to numerous stressors [20]. The causes for the initiation of cellular senescence, i.e., the stressors that induce its onset, are different, but the most commonly discussed are the effects of DNA damage, oxidative stress, oncogenic activity and chemotherapeutic toxicity [20]. In this process, cells undergo epigenetic, transcriptional, metabolic and morphological changes [8]. As long as cell damage by these factors does not cause permanent cell cycle arrest, the cell is not considered senescent [3]. Induction of cellular senescence is unidirectional and irreversible, which means that once it starts, there is no return to the initial state, and there is no way for the cell to return to any of the stages that precede the entry into senescence [3]. During this process, there is a decrease in the proliferative capacity of cells, a decrease in the number of cells and the accumulation of cellular debris, which promotes tissue damage and reduces the possibility of tissue regeneration [1,8]. It is important to understand that senescence is not a single program, such as, for example, the apoptotic program, but includes a variety of effector mechanisms [21]. These mechanisms are also found in other cellular processes, so they are not specific to cellular senescence. For example, there is the concept of reproductive senescence, oncogene-induced senescence, etc. [22]. Although senescence is most often associated with a negative aspect, as it is related to age-related diseases, it also has a significant irreplaceable non-pathological role in, for example, the process of embryogenesis [8,23,24]. The largest number of tests on the topic of cellular senescence were performed in vitro [3]. It has been shown that senescent cells have several common features: prolonged cell cycle, increased cell soma size, metabolic changes, telomere shortening and intracellular damage [3,7,25]. Gorgoulis et al. defined that the hallmarks of senescence phenotype are cell cycle withdrawal, macromolecular damage, secretory phenotype and dysregulated metabolism [26]. A prolonged cell cycle delays the entry of the cell into the phase of division thereby reducing its reproductive capacity [3,7,26]. Tests performed on human diploid fibroblasts prove this. A decrease in cell proliferative power is accompanied by an increase in size gradually during the aging process [27,28]. In addition, in order for the cell cycle to proceed smoothly, it is necessary that many metabolic pathways function in an efficient manner [4]. In cells that are in the process of senescence, significant changes occur in the metabolic pathways of almost all macromolecules [4,20]. Some of the most significant changes are an increase in the level of glycolysis, a decrease in the capacity for oxidative phosphorylation and a decrease in the level of nicotinamide adenine dinucleotide (NAD), intracellularly [29,30,31]. These changes also affect the clonal ability of the cells and their importance can be understood through the study results. In vivo research on a murine lymphoma model in which cellular senescence was initiated by chemotherapy proved that tumor regression occurs by blocking glycolysis [32]. Another study showed that the supplementation of cells with NAD precursors increases reproductive lifespan and replicative capacity [33,34]. In addition to the above, it is important to mention that senescent cells are characterized by progressive telomere shortening [6,35,36]. They represent complexes of nucleoproteins which are protecting the ends of chromosomes from the action of enzymes [37]. They are built from special tandem repeats (5′-TTAGGG-3′) and are associated with multiprotein complexes called shelterin [37]. The role of shelterin is to protect the ends of chromosomes from damage in the process of DNA metabolism, but this means that they also limit the possibility of DNA repair when this damage is done [37]. The most important protein from this group is the telomeric repeat–binding factor (TRF2) [38]. When the telomere length is significantly reduced and when they reach the so-called “critical length”, double-strand breaks occur. This triggers the DNA-damage response (DDR) [39]. As we will see later, this process is most responsible for the initiation and maintenance of senescence [8,39]. It is still not known what is the exact threshold of telomere length or the number of dysfunctional telomeres required to induce senescence [6]. A cell is considered senescent when its telomeres have reached a critical length, and given that DNA damage can only be repaired if the cells have the possibility of reproduction; when permanent cell cycle arrest occurs, the possibility of repairing the damage is lost [36].

The most important stimulus for the initiation and maintenance of senescence is DDR [8,39]. Activation of the p53 tumor-suppressor gene causes the permanent activation of this mechanism [35,40]. Double-strand breaks are the most important form of DNA damage [41]. These damages by complex mechanisms lead to cell cycle arrest and apoptosis. During the aging process, the number of such damages increases but this increase is not linear [40,42]. Cells reach their maximum while they still have the possibility of replication, that is, until they became senescent [40]. In research conducted on cell cultures, it has been proven that the factors that lead to the formation of double-strand breaks are stressors that affect cell replication and are important for the induction of reproductive senescence [43,44].

DDR has been shown to be important for the activation of oncogene-induced senescence [6,22]. In that case, certain oncogenes are activated, for example, Serrano et al. showed that rat sarcoma (Ras) or rapidly accelerated fibrosarcoma (Raf) are often involved, and the initial response to this is cell proliferation [45]. During intense proliferation and DNA replication that is why errors are more frequent [46]. For this reason, DDR activation was also more expressed in order to try to fix these errors. It is interesting that some studies have shown that inhibition of DDR, in this case, leads to inhibition of oncogene-induced senescence [47,48,49].

**Figure 2:**
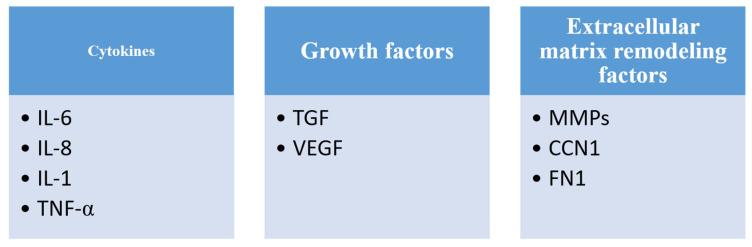
The components of SASP are numerous, but the most important ones are: interleukin (IL) 6, 8 and 1, tumor necrosis factor alpha (TNF-α), transforming growth beta (TGF), vascular endothelial growth factor (VEGF), matrix metalloproteinases (MMPs), protein CCN1 and fibronectin1 (FN1).

Another way to initiate DDR is the occurrence and registration of telomere damage and shortening. Salama et al. in their review report on the importance of telomeres and their shortening for the initiation of DDR [8]. According to research known so far, there are three functional states in which telomeres can be found: closed state, intermediate state and uncapped state [50]. In each of these three conditions, the ends of chromosomes are exposed to different influences, and in response to the damage, DDR is initiated [50]. The most important for the emergence of persistent DDR, which is important for the induction of senescence, is the intermediate state. In this state, regardless of the damage, the chromosomes retain the TRF2 protein, which, in addition to protecting against further damage, also prevents the repair of the one that has occurred up to that point, thus, starting a persistent DDR [50]. As we said before, senescent cells change at the metabolic, morphological, epigenetic and transcriptional levels, and they are also characterized by increased secretory activity [8]. The secretome represents all proteins secreted by a cell, tissue or organ. Therefore, senescent cells have a specific secretome that differs from the secretome of young cells in which senescence has not yet been induced [8]. The secretome of senescent cells includes mostly pro-inflammatory cytokines, chemokines, proteases and growth factors [51,52] (Figure 2).

The action of these molecules enables changes in the pathophysiology of cells and tissues, which are characteristic of senescence and play a role in the development of age-related diseases. Secretome proteins of senescent cells were mostly discovered by research on human diploid fibroblasts, but also by research conducted on other cells in vitro and in vivo [22,52]. They can be considered the markers of senescence, and they need to be interpreted in several different contexts [6,52]. Considering that they are also found in other cellular processes and are not specific only to senescence, it is important to interpret the combination of several markers [8,53]. The secretome of senescence cells is called by another name senescence-associated secretory phenotype or SASP. The composition of SASP depends on the cell type and the way it entered in senescence [22]. SASP is regulated on several different levels: through persistent DDR, transcriptional regulation and autocrine regulation [54]. The inflammasome, which will be described later, also proved to be a factor in the induction of SASP. Persistent DDR, which was discussed earlier, has proven to be the most important factor in SASP regulation for now. For example, the loss of ataxia telangiectasia-mutated factor (ATM), Nijmegen breakage syndrome 1 mutated gene (NBS1) or checkpoint kinase 2 (CHK2), which participate in DDR, leads to a decrease in the release of certain SASP products such as IL-6 and IL-8 [40]. These two interleukins are not only important for DDR but are also mentioned as important components of oncogene-induced senescence (OIS), which means that they are involved in the process of tumor genesis [40]. Another example is that the expression of p16 or p21 leads to the initiation of senescence without the initiation of DDR and changes in SASP in terms of reduced release of pro-inflammatory cytokines [40]. The loss of the p53 tumor-suppressor gene in human diploid fibroblasts promotes the secretion of IL-6 and the formation of DDR, which has a role in the genesis of tumors [55]. Lujambio et al. demonstrated that p53-mediated SASP in hepatic stellate cells suppresses the formation of HCC by activating special M1 macrophages [55]. This study was conducted on a mice model in which the formation of HCC was induced by chemotherapeutic agents. Some transcription factors, such as nuclear factor kappa B (NF-κβ) and CCAAT/enhancer-binding proteins β (C/EBPβ), also participate in the regulation of the release of proinflammatory components of SASP [56,57,58]. C/EBPβ has as a target the synthesis of IL-6 and its inactivation leads to a reduction in the inflammatory effect of SASP. NF-κβ and C/EBPβ also regulate the release of IL-1 and IL-8 [59]. These two transcription factors have been shown to play an important role in the OIS process [57,58].

**Figure 3:**
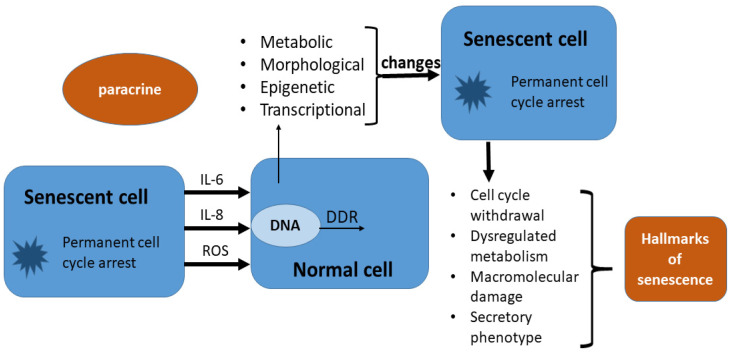
The bystander effect: senescent cells are more secretory active than non-senescent cells and paracrine can affect young cells and induce the start of the senescence process in them. This is accomplished through proinflammatory cytokines and reactive oxygen species (ROS) which cause deoxyribonucleic acid (DNA) damage and initiate DNA damage response (DDR). In this way, the normal cell enters in permanent cycle arrest or senescence. Hallmark of senescence is defined by Gorgoulis et al. [26].

The effects of SASP are numerous. They are realized in two ways: paracrine and autocrine. Paracrine effects of SASP include: pro-tumorigenesis, immunomodulation, senescence reinforcement and modulation of the tissue microenvironment [60]. Senescence reinforcement is also an autocrine effect of SASP, and it is mediated by IL-6 and IL-8 [54]. It has been proven that senescent cells are more secretory active and that paracrine can affect young cells and induce the start of the senescence process in them. That effect is called “the bystander effect” (Figure 3) [61,62,63].

It implies the harmful influence of senescent cells on younger cells through reactive forms of oxygen (ROS). They cause DNA damage and introduce non-senescent cells into permanent cell cycle arrest [61,62,63]. In this way, the proliferative capacity of cells is reduced. The existence of “the bystander effect” was proven by determining senescence markers in younger cells, and it is interesting that in epithelial cells and cancer cells, this effect more often stimulates proliferation, which is the opposite of what was previously described [64,65]. It has been proven that SASP products of human diploid fibroblasts can lead to the proliferation and transformation of premalignant epithelial cells into malignant ones [64,65]. The uniqueness, complexity and importance of SASP are also in the fact that it abounds in a multitude of effects, some of which are contradictory. For example, another paracrine effect of SASP is the stimulation of anti-tumor immunity. SASP paracrine products trigger the so-called “senescence surveillance” composed of components of the innate and adaptive immune response, which has a role in the removal of senescent cells, the suppression of tumor formation and their regression [55,63,66]. SASP components actually enable tissue infiltration by natural killer (NK) cells that then eliminate senescent and tumor cells [67,68]. In addition, individual SASP components play a role in changes in tissue morphology. For example, IL-1β, in addition to having a pro-inflammatory role, participates in the modulation of the tissue microenvironment [63]. In a study conducted by Krizhanovsky et al. on mice liver models, chronic toxic liver damage is accompanied by the activity of hepatic stellate cells in terms of proliferation and formation of profibrotic secretome [66]. This leads to the accumulation of hepatic stellate cells in which senescence is induced, and their secretome changes into a secretome similar to SASP. It is characterized by the presence of matrix-degrading enzymes, which limits the degree of liver fibrosis. Senescent hepatic stellate cells on the end are removed by the “senescence surveillance” mechanism [66].

After all, we can conclude that cellular senescence is very complex as it involves many different pathways, many molecules of different origins, enzymes and transcription factors. Therefore, it is necessary to take into account several different markers, as well as their combinations, in an attempt to detect senescence [69].

## 3. Inflammaging

As we have seen, SASP involves the production of numerous pro-inflammatory cytokines that play different roles in multiple processes that are vital for the cell [1,8,22]. Their main role is in promoting the occurrence of chronic inflammation in tissues. According to numerous studies, chronic inflammation is an irreplaceable factor in aging process and the onset of age-related diseases such as dementia, atherosclerosis, osteoporosis, cancers, metabolic syndrome and vascular diseases [70,71]. It leads to the activation of numerous proinflammatory signaling pathways. In light of the connection between cellular aging and inflammation, the term “inflammaging” was introduced [70,72,73,74,75,76]. This term was created after Fagiolo et al. showed in that peripheral blood mononuclear cells of elderly people produce a higher amount of pro-inflammatory cytokines compared to cells of younger people [77]. After that, this phenomenon was the subject of numerous studies that came to the conclusion that “inflammaging” is associated with the aforementioned increased production of pro-inflammatory cytokines in the elderly, genetic components and frequent subclinical viral infections seen in the elderly (Epstein Barr virus and cytomegalovirus infection) [77]. Inflammation as part of “inflammaging” is the so-called “sterile inflammation”, i.e., inflammation in which the existence of a pathogen that would cause it has not been proven [75,76]. It affects the tissues in multiple ways, for example, by enabling infiltration of tissue by cells of the immune system and, on the other hand, by the effect of proinflammatory cytokines that can cause phenotypic changes in previously normal cells (disruption of cellular communication, damage to the innate immune response, stimulation of angiogenesis, etc.) [78,79]. Baker et al. have shown in repeated research that by reducing the number of senescent cells, there is a reduction in SASP and the release of pro-inflammatory cytokines, thus, proving that SASP plays a role in “inflammaging” [73]. The importance of inflammation in the senescence process can also be concluded based on the fact that the complex known as “inflammasome” participates in the regulation of SASP [63]. It is a complex composed of several molecules belonging to the innate immune response. It activates caspase-1, which is responsible for the activation of the IL-1 inflammatory cascade that promotes the induction of senescence through oxidative stress and DNA damage [80]. Other pro-inflammatory cytokines similarly lead to age-related inflammation, for example, IL-6 and TNF-α [70]. Some authors mention the blockade of the inflammatory response in SASP as one of the potential therapeutic possibilities for diseases associated with senescence. Drugs with this effect are called senomorphics [81]. They lead to inhibition of SASP, but not to apoptosis of senescent cells. They achieve their effect by inhibiting the pathways responsible for the formation of SASP as well as transcription factors signal transducer and activator of transcription (STAT) protein, NF-κB and C/EBP β [82]. Senomorphics include rapamycin, everolimus, resveratrol, apigenin and ruxolitinib [82]. Some well-known drugs that are used for other indications have also shown a senomorphic effect, for example, metformin [83]. Bearing in mind that in senescence-related diseases there is an increase in the number of senescent cells, the possibility of removing these cells could eventually prevent the onset of the disease. Studies on mouse models have shown that the removal of senescent macrophages reduces the risk of atherosclerosis and that reducing the number of senescent glial cells can prevent cognitive disorders [84,85]. Drugs that have this function are called senolytics and they selectively induce apoptosis of senescent cells. Senescent cells initiate pro-survival pathways that hinder the occurrence of apoptosis; however, this mechanism enables the targeting senescent cells by senolytic drugs [86]. The first discovered senolytics are natural substances whose role was discovered in 2015, namely quercetin and dasatinib [87]. After that, research progressed and seven more classes of the mentioned drugs were discovered, some of them are: fisetin, piperlongumine, curcumin, navitoclax, ABT-737, A1331852, UBX3125, P5091, geldanamycin, tanespimycin, FOXO4-DRI, etc. They act according to different principles, some of them are kinase inhibitors, BCL-2 family inhibitors, inhibitors of MDM2/p53 interaction, Hsp90 inhibitors, p53 binding inhibitors, etc. [88].

## 4. Signs That Reveal an Aging Liver

**Figure 4:**
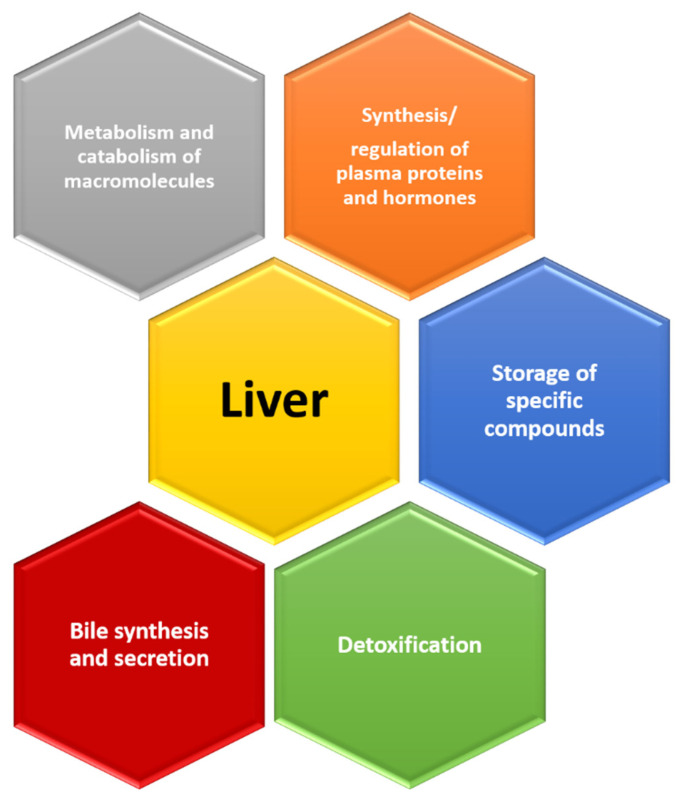
Roles of the liver.

The liver is an organ that has several important roles in our body: metabolism of carbohydrates, proteins and lipids, synthesis/regulation of plasma proteins and hormones, storage of specific compounds, detoxification, bile synthesis and secretion, catabolism of different molecules, etc. [13] (Figure 4). Changes at the level of the liver parenchyma have been extensively investigated, and the results are important also because of the possibility of transplantation of this organ in end-stage liver disease [13].

The limitations of this process are numerous and require detailed examination. Studies have shown both morphological and functional differences in the liver of older and younger people. In elderly people, the volume of the liver decreases by 20–40%, depending on the sex, and the hepatocytes of the elderly have a reduced volume, have a higher level of lipofuscin and secondary lysosomes, and polyploidy is more common in them [10,12,13,89]. In people older than 85 years, even 27% of hepatocytes show polyploidy [90]. Lipofuscin is a pigment that accumulates intracellularly and is a product of catabolism. At the end of the 19th century, it was noticed that it accumulates in the cells during aging [91,92]. It is made of proteins that have undergone oxidation, as well as lipids (triglycerides, free fatty acids, cholesterol and lipoproteins) and carbohydrates [91,92]. In its composition, lipofuscin also contains zinc, aluminum, manganese and copper, but also iron, which is considered a source of free radicals [93]. Lipofuscin today is known as a potentiator of intracellular dyshomeostasis and apoptosis, but its role is not fully understood yet. In the case of senescence, it was shown that lipofuscin potentiates the expression of the anti-apoptotic factor bcl-2, which enables the resistance of senescent cells to apoptosis [26]. Lipofuscin is considered a hallmark of cellular senescence because it is associated with accompanying phenomena such as macular degeneration [94]. This was proven by Geogakopoulou et al. by histochemical determination of lipofuscin using Sudan-Black-B (SBB) staining [95]. They examined cells in which cellular senescence was induced by stressors and replicative exhaustion in a mission model. It turned out that senescent cells as well as the tissues that contain them are stained with SBB, which proves the presence of lipofuscin in them and justifies the importance of lipofuscin as a marker of cellular senescence [95]. In a similar way, the same conclusion was reached by Kohli et al [69]. Salmonowicz and Passos mention a more precise way of registering lipofuscin in aging cells, and it involves the use of an analogue of the previously mentioned SBB called GL13. It has been shown that the combination of these two molecules has an advantage over the independent use of SBB [91]. From clinical aspect, the importance of lipofuscin is also reflected in the fact that it can have an impact on the pathogenesis of the disease like in congenital neuronal ceroid lipofuscinoses, whose course is progressive and ends lethally in the early years of life [96].

As mentioned, the process of telomere shortening is one of the keys to the process of senescence. Even in the case of liver aging, the presence of this process indicates senescence, and it has been shown that the stellate and sinusoidal cells of the liver are responsible for this phenomenon [97]. It has been proven that patients with chronic viral hepatitis have shorter hepatic telomeres compared to healthy people and that the degree of liver fibrosis in this case increases with age [98,99]. One of the studies showed an association between the degree of telomere shortening and the degree of progression of liver fibrosis in patients with chronic hepatitis C virus (HCV) infection [100]. Telomere length has been shown to be significant for the development of HCC [101]. Shorter hepatocyte telomeres have an inhibitory effect on the development of HCC [102]. Plentz et al. showed that the telomeres of HCC cells are shorter compared to the surrounding healthy cells, which means that, in this case, the expected initiation of DDR and permanent cell cycle arrest does not occur as we would expect [103].

The results of the studies at the molecular level made it possible to register senescence in liver cells. Morsiani et al. showed that in people aged between 60 and 70 years there is an increase in the expression of three types of micro ribonucleic acid (microRNA) (miR-31-5p, miR-141-3p, miR-200-3p), which is why they proposed these molecules as markers of the liver senescence process [3,104]. It was also determined that the changes present in the liver parenchyma during aging are the result of changes in the glutamine pathway and DNA methylation pattern [105,106]. IL-6, as one of the basic pro-inflammatory cytokines and SASP components, can serve as a marker of senescence of liver cells, that is, a marker of inflammation and activity of hepatocytes [107]. Interestingly, it is mostly produced by Kupffer cells of the liver, but they do not have receptors for it [108]. Hepatocytes are one of the few cells in our body that have a receptor for IL-6, which manifests its effect and induces the acute phase response, but also proliferation in order to regenerate damaged cells [108]. Cellular senescence at the level of the liver includes, in addition to Kupffer cells and hepatocytes, hepatic stellate cells and mast cells [109,110,111,112]. The consequence of chronic inflammation is the potential acceleration of the progression of liver damage, as well as the “inflammaging” process and the onset of age-related diseases [75]. Chronic inflammation has been shown to be necessary for the progressive liver dysfunction that occurs with aging. The incidence of liver disease increases with age, and also age is often an independent predictor of worse outcomes in various liver diseases [12].

## 5. Liver Diseases and Aging

### 5.1. Non-Alcoholic Fatty Liver Disease (NAFLD), Non-Alcoholic Steatohepatitis (NASH), Alcoholic Steatohepatitis (ASH) and End-Stage Liver Disease

The liver is characterized by a slow-aging process that is multifactorial and still insufficiently investigated [12]. It is known that various environmental factors and lifestyles, as well as alcohol consumption and the influence of toxic substances, lead to the development of chronic inflammation of the liver [113]. The liver initially reacts to harmful noxes with the appearance of steatosis, which over time and under the influence of numerous endogenous factors can progress to NAFLD [114,115,116,117]. Studies have shown that the frequency of developing liver cirrhosis in patients with NAFLD increases with age and that patients older than 50 years are at a higher risk for developing severe fibrosis [118,119]. NAFLD has a tendency to progress to NASH which is associated with obesity, metabolic syndrome, type 2 diabetes, insulin resistance and cardiovascular diseases [120]. The frequency of most of the mentioned conditions increases with age [3]. In the case of alcohol abuse, ASH occurs, which, if not treated, like NASH, can lead to progressive fibrosis with the development of liver cirrhosis [121,122,123]. Age is an independent predictor of worse outcomes in patients with ASH [124].

The importance of changes in liver function during aging can also be seen in light of the need for liver transplantation in patients with end-stage liver disease. Considering the increase in the number of these patients and the growing need for transplantation, new studies were conducted to give information on the utilization of the organs of old and very old donors, especially the possibility of transplanting their organs to younger patients [125,126]. It was shown that when transplanting an organ from an old donor to a young recipient, the expression of the three previously mentioned microRNAs does not change [104]. The reverse has not been proven with success. This data indicates that the aging phenotype is more easily transmitted than the younger phenotype, at least in the case of liver transplantation [104]. Numerous studies have shown that the biological and chronological age of a person does not have to match, as well as that the biological and chronological age of the liver can be different [127]. This fact provides less restrictions when choosing the donor and the recipient, but prior extensive tests are necessary. According to all the above, if there is no significant inflammatory response of the liver tissue and its harmful consequences are avoided, the liver is an organ that ages more slowly than other organs and has a greater regeneration capacity compared to them. The regenerative capacity of the liver is age-related [128,129]. Research conducted on a rat model in which partial hepatectomy was performed showed that complete hepatic restoration in older experimental animals is slower than in younger ones [130].

### 5.2. Viral Hepatitis

Hepatitis A virus (HAV) is an RNA virus that is highly contagious and is transmitted by the fecal–oral route (Table 1). The severity of the clinical presentation depends on age, so middle-aged and elderly people more often show a more severe presentation of this infection [131,132]. Elderly people are at a higher risk of developing acute liver failure and have higher mortality, and it has been proven that age is an independent predictor of poor outcomes [131,132,133]. For this reason, it is advised to vaccinate elderly people with chronic liver disease, even those with end-stage liver disease [134].

Hepatitis B virus (HBV) is a DNA virus that belongs to the Hepadnaviridae family and is spread by parenteral or sexual contact [135] (Table 1). Given that it is associated with risky behavior, including intravenous drug use and risky sexual contact, the incidence of acute infection is lower in older age [136]. Chronic HBV infection is most common in older patients, and differences in incidence exist in relation to ethnicity [132,136]. Patients with chronic HBV infection are at higher risk for developing HCC; therefore, adequate and timely treatment is important [137]. It is interesting that some authors noticed a connection between HBV infection, cellular senescence and the occurrence of HCC [137]. In patients with chronic HBV infection, as in other people, part of the liver cells enters senescence over time. There is an increase in the number of senescent cells which, as a basic feature, have a reduced proliferative capacity. That characteristic reduces the possibility of tumorigenesis namely the possibility for the development of HCC. On the other hand, in the process of senescence there are changes in the microenvironment of cells and the activation of the immune “senescence surveillance” mechanism which in conditions of chronic HBV infection may be insufficient to remove the required number of senescent and precancerous cells and, in that way, potentiates the development of HCC [137]. Given the serious consequences of this infection, a prevention strategy was developed and it includes the vaccination of infants, children, adolescents and adults who are at increased risk for infection, promoting protected sexual contact and healthy lifestyles in order to reduce the incidence of intravenous drug use [138,139].

Hepatitis C virus (HCV) is an RNA virus from the Flaviviridae family [140,141]. Like HBV, it is also transmitted by parenteral or sexual contact [135,141] (Table 1). Intravenous drug use is still mentioned as the biggest risk factor [132]. The incidence of acute infection decreases with age but, considering that the world’s population is getting older, the incidence is expected to increase even in this age group [132]. Risk factors for the progression of chronic HCV infection are older age, chronic alcohol abuse, co-infections and male gender [142]. Elderly patients with chronic HCV infection show more marked progression of fibrosis, more frequent development of cirrhosis and HCC [132]. Gerontological patients represent a challenge in the treatment of HCV infection, and therapeutic options are significantly limited [143,144]. Studies conducted on elderly patients are rare and generally do not include patients older than 65 years [145]. Interferon (IFN) monotherapy has been shown to reduce mortality in patients over 60 years of age, and patients with chronic HCV infection and liver cirrhosis treated with IFN have a reduced risk of HCC compared to those who were not treated [146]. One of the studies indicated that the effectiveness of the use of pegylated IFN and ribavirin in older patients is lower than in younger patients, while there are data showing that the difference in the response of older patients and patients younger than 60 years is not statistically significant [147,148]. 

### 5.3. Hepatocellular Carcinoma (HCC)

Numerous studies so far have shown that the appearance of HCC is related to old age; that is, the incidence of this carcinoma increases with age, especially in the population over 75 years old [149,150]. Gerontological patients are at an increased risk due to changes in physiological processes and metabolism, which also affect therapeutic options in diagnosed patients [151]. Despite this, there are still not enough studies in the sphere of surgical and medical treatment of HCC in elderly patients [14]. Elderly patients with HCC are mostly negative for hepatotropic viruses, and otherwise more often have HCV infection [152]. Older patients with HCC are more likely to be female, which can be attributed to the longer life expectancy of women [153]. In this population, NASH-related-HCC is detected more often than in younger people [14,154]. Liver fibrosis is less pronounced in patients who are older; therefore, they have smaller HCC nodules compared to younger patients [155,156]. Surgical treatment of HCC represents the possibility of a complete cure for the patient, and the progress in technique and technology has improved the outcome in elderly patients [157,158]. Radiofrequency ablation (RFA) is a therapeutic treatment for HCC that causes thermal injury to carcinoma cells resulting in their coagulation necrosis [159]. Elderly patients are often candidates for RFA due to comorbidities [14]. Two studies compared 3- and 5-year overall survival of older and younger patients and came to the conclusion that there is no significant difference between these groups, while the results of the study conducted by Nishikawa et al. are contradictory [160,161,162]. Transarterial chemoembolization (TACE) is a therapeutic option that is applied when the tumor is unresectable and belongs to palliative therapy [14]. Recent data support the safety and efficacy of TACE in elderly patients [163,164] (Table 2).

## 6. Conclusions

Bearing in mind all of the above and the significant share of elderly people in the world’s population, as well as the fact that the diseases that are often encountered in this group carry with them numerous complications, new research on the topic of senescence is necessary. The results so far show that the changes at the cellular level are complex and that the senescence phenotype can spread to the surrounding non-senescent cells, thereby causing a progressive detheorization of tissue and organ function. The predominance of the pro-inflammatory character of SASP is characterized by chronic inflammation, which has multiple harmful effects. In addition, the role of SASP in the genesis of tumors has been proven, which makes the research of cellular senescence even more important. The liver, as an organ that, according to the results of some studies, ages more slowly and regenerates faster than other organs also undergoes significant changes in the aging process. As a result of this, but also with the action of other factors (alcohol, lifestyle habits, diet...), chronic diseases occur and their progression can lead to end-stage liver disease and the need for transplantation. Given the limitations in the application of certain therapeutic modalities in the elderly, it is necessary to conduct new studies that would enable early detection and prevention of the onset of age-related diseases, as well as the development of new therapeutic options that would be safe and effective for elderly patients.

## Figures and Tables

**Table 1:** Hepatotropic viruses.

Virus	Genetic Material	Family	Transmission	Complication
Hepatitis A (HAV)	RNA	*Picornaviridae*	Feco-oral	Acute liver failure
Hepatitis B (HBV)	DNA	*Hepadnaviridae*	Parenteral or sexual	Liver cirrhosis, HCC
Hepatitis C (HCV)	RNA	*Flaviviridae*	Parenteral or sexual	Liver cirrhosis, HCC

**Table 2:** Hepatocellular carcinoma in elderly patients.

Hepatocellular Carcinoma (HCC) Facts	Treatment
Elderly patients are mostly negative for hepatotropic viruses.	Surgical
Elderly patients are more likely to have HCV than other hepatotropic viruses.	Radiofrequency ablation (RFA)
Elderly patients are more likely to be female.	Transarterial chemoembolization (TACE)
NASH-related-HCC occurs more often in elderly than in young people.	
Liver fibrosis is less pronounced in elderly patients.	

## Data Availability

Not applicable.
